# RhoGTPase Regulators Orchestrate Distinct Stages of Synaptic Development

**DOI:** 10.1371/journal.pone.0170464

**Published:** 2017-01-23

**Authors:** Samuel Martin-Vilchez, Leanna Whitmore, Hannelore Asmussen, Jessica Zareno, Rick Horwitz, Karen Newell-Litwa

**Affiliations:** 1 Department of Cell Biology, University of Virginia School of Medicine, Charlottesville, VA, United States of America; 2 Department of Anatomy and Cell Biology, Brody School of Medicine, East Carolina University, Greenville, NC, United States of America; UPR 3212 CNRS -Université de Strasbourg, FRANCE

## Abstract

Small RhoGTPases regulate changes in post-synaptic spine morphology and density that support learning and memory. They are also major targets of synaptic disorders, including Autism. Here we sought to determine whether upstream RhoGTPase regulators, including GEFs, GAPs, and GDIs, sculpt specific stages of synaptic development. The majority of examined molecules uniquely regulate either early spine precursor formation or later maturation. Specifically, an activator of actin polymerization, the Rac1 GEF β-PIX, drives spine precursor formation, whereas both FRABIN, a Cdc42 GEF, and OLIGOPHRENIN-1, a RhoA GAP, regulate spine precursor elongation. However, in later development, a novel Rac1 GAP, ARHGAP23, and RhoGDIs inactivate actomyosin dynamics to stabilize mature synapses. Our observations demonstrate that specific combinations of RhoGTPase regulatory proteins temporally balance RhoGTPase activity during post-synaptic spine development.

## Introduction

RhoGTPases are molecular switches that orchestrate various signaling pathways and are best known for being master regulators of actin cytoskeleton polymerization and organization [1]. Recently, they have emerged as crucial regulators of neuronal development, including dendritic arborization, growth cone development, axon guidance, and post-synaptic spine morphogenesis underlying excitatory neurotransmission [2–4]. During normal synaptic development, the small RhoGTPase, Rac1, promotes the formation of filopodia-like spine precursors [5–8] that subsequently mature through RhoA/ROCK-dependent myosin II activation into polarized mushroom-shape spines [9,10]. Further excitatory stimulation associated with long-term potentiation leads to Rac1-driven spine head expansion [6,11].

Through these mechanisms RhoGTPases also impact learning and memory. Altered RhoGTPase signaling leads to abnormal spine morphology and synaptic development and appears to contribute to the pathology of neuronal disorders, such as Autism Spectrum Disorders and non-syndromic mental retardation, as well as neurodegenerative disorders, like Alzheimer’s disease (AD) [12–16].

RhoGTPases are activated by Guanine Exchange Factors (GEFs) and inactivated by GTPase-Activating Proteins (GAPs), while Guanine Dissociation Inhibitors (GDIs) attenuate RhoGTPase signaling by binding and sequestering the inactive GDP-bound state in the cytosol [17]. Several of these upstream RhoGTPase regulatory proteins are implicated in neurodevelopmental disorders. For example, mutations in the RhoA-GAP OLIGOPHRENIN-1 result in non-syndromic mental retardation [16], through glutamatergic dysfunction, preventing dendritic spine development and synapse maturation [15,18]. Furthermore, RhoGTPases are disproportionately represented in copy number variants associated with Autism and schizophrenia, further highlighting the fundamental developmental role of RhoGTPases in shaping proper neuronal connections [19,20].

Despite a fundamental role for RhoGTPase signaling in neurons, how and when these signaling pathways are activated is not known. In this present study, we explored whether RhoGTPase regulators exhibit stage-specific roles in synaptic development. Our results suggest that RhoGTPase regulators function temporally at discrete stages of synaptic development. Individual regulators orchestrate actomyosin dynamics that support post-synaptic spine morphogenesis in either early spine precursor formation or later spine maturation. In addition to demonstrating that specific molecules uniquely remodel synaptic architecture at distinct developmental periods, this study also suggests the feasibility of targeted therapeutic intervention of actomyosin regulation during synaptic plasticity.

## Results

### Expression of synaptic RhoGTPase regulatory proteins in neuronal development

**Fig 1:**
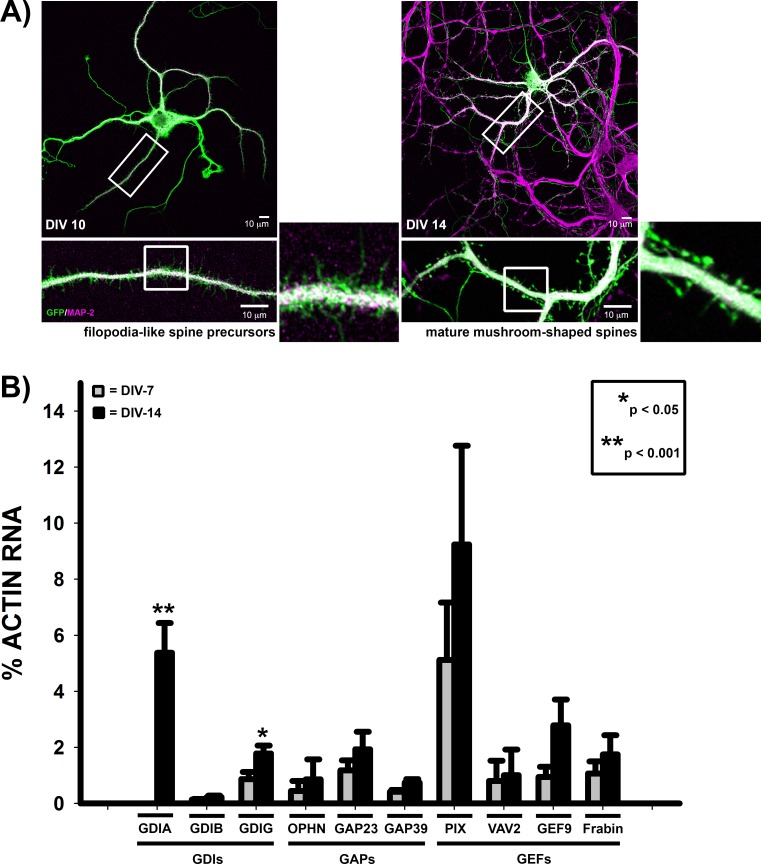
Expression of RhoGTPase regulators during synapse development. **A)** Primary rat hippocampal neurons expressing GFP were stained for the dendritic marker, MAP-2 (MAP-2). At ~1 week in culture (days in vitro, DIV-10), filopodia-like spine precursors extend from the dendritic shaft. However, after 2 weeks, spines begin to mature into a polarized mushroom-shape, characterized by a bulbous spine head atop a thin spine neck. **B)** Real-Time RT-PCR of RhoGTPase regulators from RNA harvested from primary rat hippocampal neurons grown for either 1 or 2 weeks in culture (DIV-7, grey bars; DIV-14, black bars). RNA expression levels are expressed as a percentage of actin RNA. (n ≥ 5 neuron cultures for each time point; p<0.001 for *Arhgdia* expression at DIV-7 vs DIV-14 (Mann-Whitney Rank Sum Test) and p = 0.033 for *Arhgdig* expression at DIV-7 vs DIV-14 (t-test); RNA expression levels of all other regulators was not statistically significant between DIV-7 and DIV-14.

While previous studies have largely focused on a particular stage of synaptic development, we sought to determine how RhoGTPase regulators function throughout synaptic development by evaluating their contribution to both early development, when immature filopodia-like spine precursors form, and later synapse formation, when spines mature into a mushroom-shaped morphology. These stages of synapse development are observed *in vitro* with cultured rat hippocampal neurons; they exhibit robust spine precursor formation at approximately 1 week in culture and spine maturation after 2–3 weeks in culture (Fig 1A) [9,21,22]. We used shRNA to acutely downregulate expression of select RhoGTPase regulators at these distinct stages of synaptic development (S1 Fig). We selected known synaptic RhoGTPase regulatory proteins, β-PIX, a GEF [5,23,24], and OLIGOPHRENIN-1, a GAP protein that is mutated in non-syndromic mental retardation [15,18]. We also included the three mammalian RhoGDI family members (α, β, and γ)[25], to develop a broader and more holistic picture of RhoGTPase regulation during synaptic development. All of these RhoGTPase regulators are either mutated or have copy number variants associated with known synaptic disorders, including non-syndromic mental retardation and Autism Spectrum Disorders (ASD) (Table 1), although their specific contribution to disease pathology may not yet be determined. As a negative control for excitatory synapse development associated with spines, we included ARHGEF9, a regulator of gephyrin clustering and inhibitory synapse formation [26,27].

**Table 1:** Synaptic Regulators of RhoGTPase Signaling.

	RhoGTPase Target	Proteome ID	Known Synaptic Function(s)	Neuronal Disease Association	Chromosome Location	Autism-Associated Copy Number Variants*
***GEFs***						
**FRABIN (FDG4)**	**Cdc42** [53]	[28]	**• Unknown**	**Mutated in Charcot-Marie Tooth** [37,38]	**12p11.21 (32,655,040–32,798,983)**	**• Deletion in Autism with Scoliosis Case [54]** **• 6 Duplications [55]** **• 4 Duplications and 1 Deletion [56]** **• 1 Reported Duplication in each publication [57,58]** **• 1 Deletion [54]**
**ARHGEF9 (COLLYBISTIN)**	**Cdc42** [59]	[31]	**• Promotes inhibitory synapse formation through gephyrin clustering [26,27]**	**X-linked Mental Retardation** [60,61]	**Xq11.1** (62,854,848–63,005,426)	**• 2 Duplications and 1 Deletion [56]**
**ARHGEF7 (β-PIX)**	**Rac** [62]	[28,31]	**• Promotes synaptic vesicle recruitment [63]** **• Increases synaptic Rac activity, resulting in increased dendritic protrusions [5,23]**	**Mutations in** β**-PIX isoform (on X Chromosome) result in non-syndromic mental retardation** [64]	**13q34 (111,767,624–111,958,081)**	**• 24 Deletions and 1 Duplication [65]** **• 16 Deletions and 16 Duplications [56]** **• 1 Deletion and 1 Duplication [55]** **• 1 Reported Deletion in each publication [57,58,66]** **• 1 Duplication and 1 Unspecified CNV Reported [67]** **• 1 Duplication [19]**
**VAV2**	**Rac (can also regulate Cdc42 and RhoA *in vitro*)** [68]	[31]	**• Promotes dendritic development [69,70]** **• Activated in Response to BDNF and increases spine head size [71]**	**None Reported**	**9q34.1** (136,627,016–136,857,726)	**• 12 Duplications [56]** **• 1 Reported Duplication in each publication [72,73]** **• 1 Deletion [74]** **• 1 Deletion [19]**
***GAPs***						
**ARHGAP23**	**• unknown**	[32]	**• Unknown**	**None Reported**	**17q12 (36,584,719–36,668,627)**	**• 1 Reported Duplication in each publication** [56,75] • **1 Duplication and 1 Deletion** [65]
**OLIGOPHRENIN-1**	**RhoA**	**N/A**	**• Regulates activity-dependent strengthening of excitatory synapses through interaction with Homer [76]** **• Regulates spine length and maturation [15,18]**	**X-linked Mental Retardation** [16]	**Xq12 (67262186–67653299**	**• 36 Duplications and 11 Deletions** [56] • **3 Duplications** [77] • **1 Reported Duplication in each publication** [78–80] • **1 Mosaic Duplication** [74] • **1 Reported Deletion in each publication** [57,81]
***GDIs***						
**ARHGDIA (RHOGDI α)**	**RhoGTPases**	**N/A**	**• Unknown**	**None, but is mutated in Nephrotic Syndrome** [82]	**17q25.3 (79825597–79829282)**	**• 3 Duplications** [65] • **1 Duplication** [83] • **1 Duplication** [19]
**ARHGDIB (RHOGDI β)**	**RhoGTPases**	**N/A**	**• Unknown**	**None Reported**	**12p12.3 (15094950–15114562)**	**• 4 Duplications and 1 Deletion** [56] • **2 Reported Deletions in each publication** [84,85] • **1 Deletion** [86] • **1 Reported Duplication in each publication** [57,58,73,87]
**ARHGDIG (RHOGDI γ)**	**RhoGTPases**	**N/A**	**• Unknown**	**None Reported**	**16p13.3 (330606–333003)**	**• 13 Duplications and 1 Deletion** [65] • **5 Duplications and 4 Deletions** [56] • **1 Reported Deletion in each publication** [55,88–90] • **1 Duplication** [57]

* In most cases, CNVs were identified through SFARI gene and include CNV reports that include all or part of the gene. In certain cases, Autism is not the primary patient diagnosis.

We also identified and included potential novel regulators of synaptic RhoGTPase activity by screening published synaptic proteomes [28–32] for targets containing either the RhoGAP domain or possessing GEF characteristics, e.g., a Dbl Homology (DH) domain followed by the Pleckstrin Homology (PH) domain [33]. This search revealed potential RhoGTPase regulatory proteins whose role in synaptic development is understudied or completely unknown. These include the GEFs, FRABIN/FGD4 and VAV2, as well as a GAP, ARHGAP23 (Table 1).

Using Real-Time RT-PCR, we assayed for mRNA expression in primary rat hippocampal neurons cultured for either 1 or 2 weeks (Fig 1B). Rat hippocampal neurons express all of the selected RhoGTPase regulators, except for RhoGDI-β, which is predominantly found in hematopoietic cells [34], and was therefore incorporated as an additional negative control. Strikingly, the Rac1 GEF, β-PIX, exhibited the highest levels of expression at both time points. However, only Rho GDI-α ανδ-γ significantly increased during neuronal maturation, suggesting a specific role for RhoGDIs in synapse maturation.

### The Rac1 GEF, β-PIX, drives spine precursor formation

**Fig 2:**
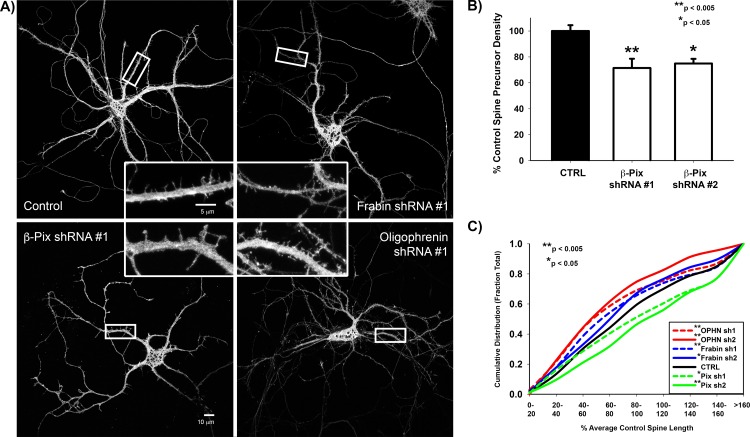
Regulators of spine precursor formation and elongation. **A)** Representative Images of GFP-expressing DIV-9 neurons transfected with the indicated shRNA targeting sequence for 72 hours. **B)** β*-pix* shRNA targeting sequences significantly decrease spine precursor density in DIV-9/10 neurons. Spine density is expressed as the percentage of the average control spine density. n = 24 control neurons, 15 β-PIX shRNA #1 neurons, and 7 β-PIX shRNA #2 neurons; p = 0.001 for Control vs β*-pix* shRNA #1 (t-test) and p = 0.007 for Control vs β*-pix* shRNA #2 (t-test). **C)** Cumulative distribution plot of spine length in DIV-9/10 primary rat hippocampal neurons co-expressing GFP and the indicated shRNA targeting sequence. Spine length is expressed as a percentage of the average control spine length. shRNAs against either the RhoA-GAP, *Oligophrenin-1*, or Cdc42-GEF, *Frabin*, significantly decrease spine length, whereas shRNAs against β*-pix* significantly increase spine length. n = 1114 control, 680 *Oligophrenin-1* shRNA #1, 115 *Oligophrenin-1* shRNA #2, 613 *Frabin* shRNA #1, 393 *Frabin* shRNA #2, 465 β*-pix* shRNA #1, and 166 β*-pix* shRNA #2 spines; p = 0.005 for Control vs β*-pix* shRNA #1 (Mann-Whitney Rank Sum Test), p < 0.001 for Control vs β*-pix* shRNA #2 (Mann-Whitney Rank Sum Test), p = 0.001 for Control vs *Frabin* shRNA #1 (Mann-Whitney Rank Sum Test), p = 0.01 for Control vs *Frabin* shRNA #2 (Mann-Whitney Rank Sum Test), p < 0.001 for Control vs *Oligophrenin-1* shRNA #1 (Mann-Whitney Rank Sum Test), p < 0.001 for Control vs *Oligophrenin-1* shRNA #2 (Mann-Whitney Rank Sum Test).

To assess the role of specific GEFs and GAPs in spine precursor formation and elongation during early stages of neuronal development, we performed acute shRNA knockdowns at approximately 1 week in culture (DIV6/7) and assessed spine morphology and density after 72 hours (DIV9/10). At one week in development, control neurons exhibit an abundance of immature filopodia-like spine precursors with very few mature spines (Fig 1A). These immature spine precursors are highly dynamic, allowing them to contact a pre-synaptic partner [9,22,35]. We therefore hypothesized that decreased expression of activators of actin polymerization, such as the Rac1 GEF, β-PIX, would reduce both spine precursor density and length. In support of this hypothesis, shRNA sequences targeting β*-Pix* decreased the density of spine precursors (Fig 2A and 2B).

**Fig 3:**
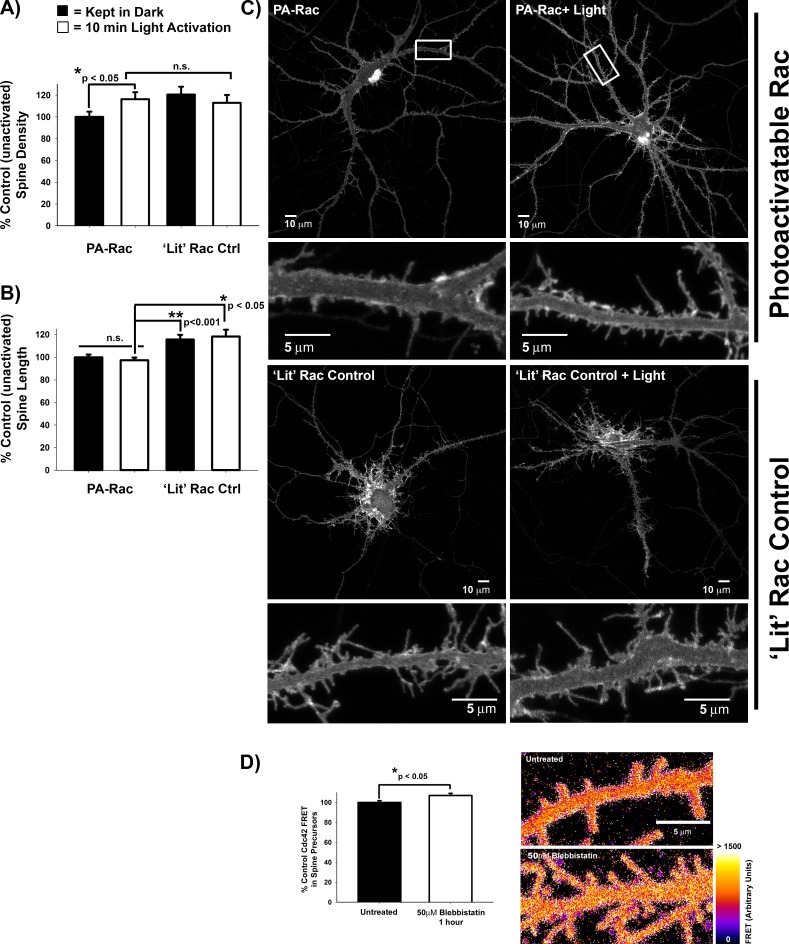
Rac drives spine precursor formation, while myosin-II and Cdc42 activity regulate spine length. **A)** Rac1 photoactivation increases spine precursor formation. DIV14-21 primary rat hippocampal neurons expressing either photoactivatable Rac1 or as a positive control, constitutively activated Rac1 (‘Lit’ PA-Rac), were kept in dark (black bars) or exposed to room lighting for 10min (white bars). The resulting spine density is expressed as percent control unactivated PA-Rac-expressing neurons. n = 24 PA-Rac neurons kept in dark, 23 PA-Rac light-exposed neurons, 8 ‘lit’ PA-Rac neurons kept in dark, and 9 ‘lit’ PA-Rac light-exposed neurons; p = 0.047 PA-Rac dark vs light-acivated (t-test). **B)** Acute Rac1 photoactivation does not affect spine length, unlike constitutive Rac1 activity (‘Lit’ control). **C)** Representative images of neurons expressing either photoactivable Rac1 (PA-Rac1, top panel) or the constitutively active ‘lit’ Rac1 control (bottom panel) that were either kept in the dark (left panel) or exposed to room lighting for 10 min (light-activated, right panel). **D)** DIV-9/10 primary rat hippocampal neurons transfected with WT Raichu Cdc42 were treated with 50μM Blebbistatin for 1 hour or left untreated. FRET was calculated as the ratio of FRET signal to CFP donor signal. Blebbistatin treatment increases Cdc42 activity by ~7%. n = 50 spine precursors each for untreated and Blebbistatin-treated, p = 0.016 (t-test).

By contrast, we did not observe consistent, significant changes in spine density with shRNAs against the other GEFs or GAPs identified in Table 1 (data not shown). This is consistent with a role for Rac1 in the formation of immature filopodia-like spine precursors [5–8]. We confirmed this role for Rac1 in the formation of filopodia-like spine precursors using a genetically encoded photoactivatable Rac1 (PA-Rac) [36], which increased formation of spine precursors along the dendritic shaft after acute photoactivation (Fig 3A and 3C and S1 Video). Unexpectedly, shRNAs against β*-Pix* did not decrease spine length, but resulted in increased length (Fig 2A and 2C), similar to expression of dominant negative Rac1T17N [6]. Consistent with this finding, while chronic Rac1 activation increased spine length (Fig 3B and 3C ‘Lit’ Rac) acute Rac1 photoactivation did not alter spine length (Fig 3B and 3C PA-Rac). Together, these studies highlight a preferential role for Rac1 in initiating spine precursor formation.

### Balanced RhoA and Cdc42 activities regulate spine precursor elongation

By contrast, shRNAs against the RhoA GAP, *Oligophrenin-1*, decreased spine length (Fig 2A and 2C) as previously described for spines in the CA1 region of rat hippocampal slices [18]. Though primarily known for its role in myelination and Charcot-Marie Tooth Disease [37,38], shRNAs against the CDC42 GEF, *Frabin*, also decreased spine length (Fig 2A and 2C). We did not observe consistent, significant changes in spine precursor length with shRNAs against the other GEFs or GAPs identified in Table 1 (data not shown). These results lead us hypothesize that competition between RhoA-driven myosin II contractility and Cdc42-mediated actin polymerization may regulate spine precursor elongation.

To test whether Cdc42 activity is negatively regulated by myosin II-mediated contractility, we measured Cdc42 activity in spine precursors using a FRET biosensor, Raichu-Cdc42 [39], following treatment with the non-muscle myosin II (NMII) inhibitor, blebbistatin. Consistent with previous results, NMII inhibition resulted in spine elongation [9,40]. Increased Cdc42 activity accompanied the spine elongation (Fig 3D), thus confirming that NMII negatively regulates Cdc42-mediated elongation to determine spine precursor length.

### ARHGAP23 promotes spine maturation

**Fig 4:**
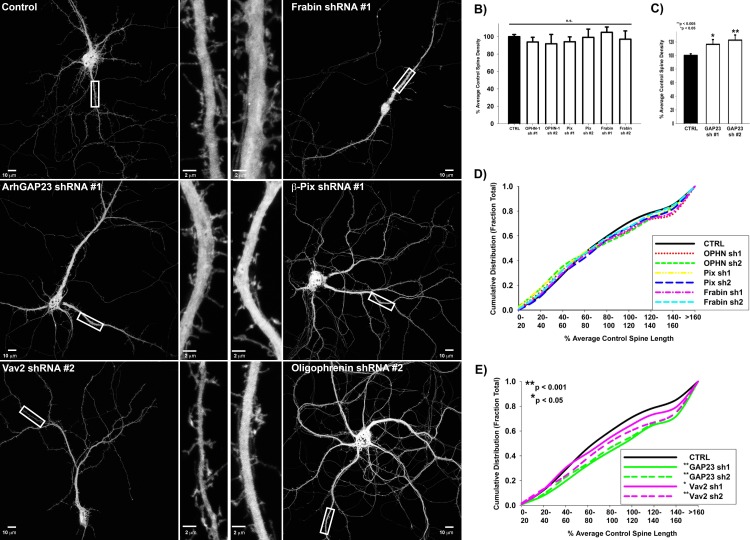
Regulators of spine maturation are distinct from regulators of spine precursor formation. **A)** Representative Images of GFP-expressing DIV-16 neurons transfected with the indicated shRNA targeting sequence for 48 hours. **B)** Regulators of spine precursor formation, OLIGOPHRENIN-1 (OPHN-1), β-PIX, and FRABIN, do not alter spine density later in synaptic development (DIV-16). Spine density is expressed as the percentage of the average control spine density. n = 44 control, 16 *Ophn-1* shRNA #1, 5 *Ophn-1* shRNA #2, 17 β*-pix* shRNA #1, 7 β*-pix* shRNA #2, 15 *Frabin* shRNA #1, 8 *Frabin* shRNA #2 neurons (Spine density was not significantly different from control as determined by t-test, except for β*-pix* shRNA #1 which was determined by Mann-Whitney Rank Sum Test). **C)** *Arhgap23* shRNAs significantly increase spine density later during synaptic development (DIV-16). n = 44 control (same as B), 22 *Arhgap23* shRNA #1, and 12 *Arhgap23* shRNA #2 neurons; p = 0.02 for Control vs *Arhgap23* shRNA #1 (Mann-Whitney Rank Sum Test), p = 0.002 for Control vs *Arhgap23* shRNA #2 (Mann-Whitney Rank Sum Test). **D)** Regulators of spine precursor formation, OLIGOPHRENIN-1 (OPHN-1), β-PIX, and FRABIN, do not alter spine length later in synaptic development (DIV-16) neurons. Cumulative distribution plot of spine length in DIV-16 primary rat hippocampal neurons co-expressing GFP and the indicated shRNA targeting sequence. Spine length is expressed as a percentage of the average control spine length. n = 3273 control, 651 *Ophn-1* shRNA #1, 130 *Ophn-1* shRNA #2, 729 β*-pix* shRNA #1, 449 β*-pix* shRNA #2, 556 *Frabin* shRNA #1, 688 *Frabin* shRNA #2 spines (Spine length was not significantly different from control as determined by Mann-Whitney Rank Sum test). **E)** *Arhgap23* and *Vav2* shRNAs significantly increase spine length later in neuronal development (DIV-16). n = 3273 control (same as D), 1207 *Arhgap23* shRNA #1, 1182 *Arhgap23* shRNA #2, 962 *Vav2* shRNA #1, and 551 *Vav2* shRNA #2 spines; p < 0.001 for Control vs *Arhgap23* shRNA #1 (Mann-Whitney Rank Sum Test), p < 0.001 for Control vs *Arhgap23* shRNA #2 (Mann-Whitney Rank Sum Test), p = 0.006 for Control vs *Vav2* shRNA #1 (Mann-Whitney Rank Sum Test), p < 0.001 for Control vs *Vav2* shRNA #2 (Mann-Whitney Rank Sum Test).

While actin polymerization drives spine precursor formation, RhoA/ROCK-mediated myosin II activation is necessary for spine maturation into a polarized mushroom-shape [9,10,41]. Furthermore, RhoA and Rac1 mutually inhibit each other’s activity [42], even at synapses [10], leading us to hypothesize that spine maturation might be regulated by either activation of RhoA and/or inactivation of Rac1. To assess this, we performed acute shRNA knockdowns of specific regulators after 2 weeks in culture (DIV14) and assessed spine morphology and density after 48 hours (DIV16) (Fig 4). Regulators of spine precursor formation, including activators of actin polymerization, β-PIX and FRABIN, and OLIGROPHENIN-1, a GAP that inactivates RhoA-driven NMII activation, did not affect spine length or density during spine maturation (Fig 4A, 4B and 4D). Interestingly, shRNAs against the GEF, *Vav2*, consistently resulted in increased spine length (Fig 4A and 4E). However, we observed the most prominent effect on mature spine morphology and density with shRNAs toward *Arhgap23*, a putative GAP identified by our in-silico screen. shRNAs against *Arhgap23* increased spine density and resulted in immature spine morphologies with increased spine length similar to filopodia-like spine precursors (Fig 4A, 4C and 4E), leading us to hypothesize that ARHGAP23 may function to inactivate Rac-driven actin polymerization.

### ARHGAP23 is a novel Rac1 GAP

**Fig 5:**
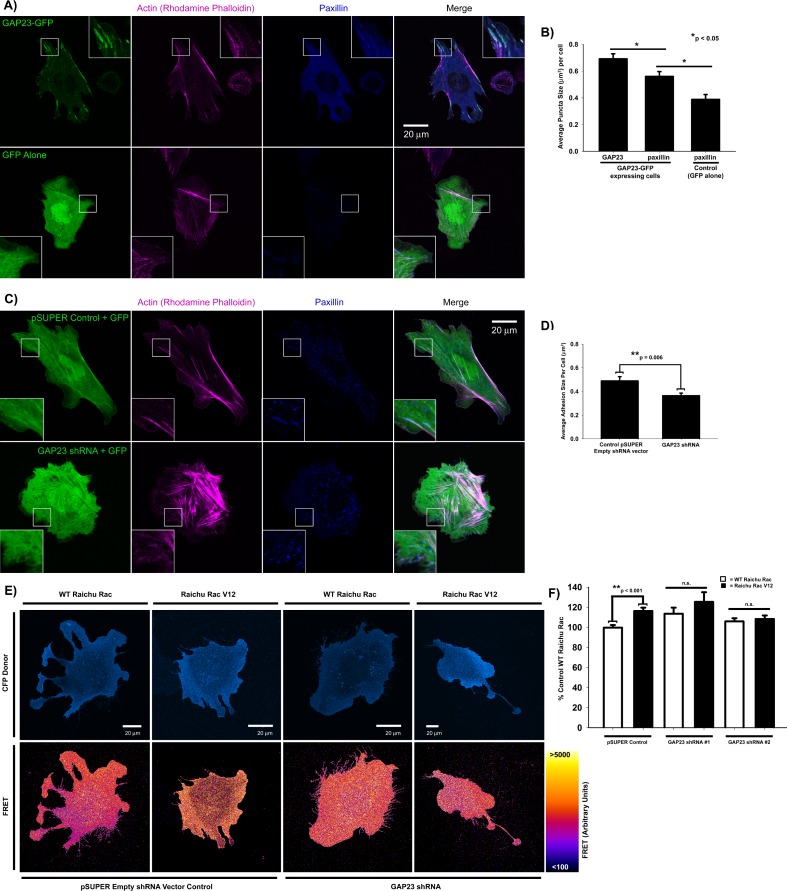
ARHGAP23 is a novel Rac GAP that regulates adhesion maturation. **A)** Representative images of ARHGAP23-GFP or GFP control CHO.K1 cells plated on fibronectin and stained for the adhesion marker, paxillin, and actin filaments (rhodamine phalloidin). **B)** Quantification of ARHGAP23 puncta size (n = 44 cells) in ARHGAP23 GFP-expressing CHO.K1 cells and paxillin puncta size in either ARHGAP23 GFP-expressing CHO.K1 cells (n = 35 cells) or control CHO.K1 cells (n = 12 cells); p = 0.015 for GAP23 vs paxillin puncta size in GAP23 GFP-expressing CHO.K1 cells (Mann-Whitney Rank Sum Test), p = 0.012 for paxillin puncta size in GAP23 GFP-expressing vs control CHO.K1 cells (Mann-Whitney Rank Sum Test). **C)** Representative images of CHO.K1 cells transfected with GFP and either control empty pSUPER vector or ARHGAP23 shRNA and plated on fibronectin. Cells were stained for the adhesion marker, paxillin, and actin filaments (rhodamine phalloidin). **D)** Quantification of adhesion size in control (n = 24 cells) or *Arhgap23* shRNA (n = 25 cells) CHO.K1 cells; p = 0.006 (Mann-Whitney Rank Sum Test). **E)** Ratiometric FRET images of control or *Arhgap23* shRNA CHO.K1 cells co-transfected with the WT Raichu Rac FRET probe or constitutively active control, Raichu Rac V12, and plated on fibronectin. The top panel shows the intensity of the CFP donor of the FRET probe in each cell. **F)** Quantification of FRET intensity in control or *Arhgap23* shRNA cells expressing Raichu Rac probes. n = 31 control WT Raichu Rac, 24 control Raichu Rac V12, 11 *Arhgap23* shRNA #1 WT Raichu Rac cells, 7 *Arhgap23* shRNA #1 Raichu Rac V12 cells, 18 *Arhgap23* shRNA #2 WT Raichu Rac cells, 13 *Arhgap23* shRNA #2 Raichu Rac V12 CHO.K1 cells; p < 0.001 for WT Raichu Rac vs Raichu Rac V12 in control CHO.K1 cells (t-test), but WT Raichu Rac is not statistically different from Raichu Rac V12 when CHO.K1 cells are transfected with either *Arhgap23* shRNA sequence (t-test).

ARHGAP23 is a novel RhoGTPase regulator identified *in silico* [43], but its cellular function is unknown. To determine whether it indeed functions as a Rac1 GAP, we used migratory CHO.K1 cells to readily visualize the effects of shRNA downregulation or expression on actin filament bundle organization and adhesion maturation, properties regulated by RhoGTPases. In migratory cells, Rac1-mediated actin polymerization contributes to the formation of the leading edge [17]. Similar to neurons, CHO.K1 cells also express ARHGAP23 (data not shown). ARHGAP23 knockdown resulted in extensive lamellipodia formation and nascent adhesions (Fig 5C and 5D). Conversely, cells expressing GFP-tagged ARHGAP23 exhibited increased adhesion maturation (Fig 5A and 5B, compare adhesions in control cells of S2 Video with adhesion maturation in a GAP23-GFP-expressing CHO.K1 cells in S3 Video). These results are consistent with a corresponding increase in RhoA-mediated actomyosin bundle formation in response to Rac1 inactivation [1,44,45]. A Rac FRET biosensor [39] confirmed that shRNA against ARHGAP23 increased Rac1 activity to levels indistinguishable from constitutively active Raichu Rac V12 (Fig 5D and 5E), demonstrating that ARHGAP23 functions as a novel Rac1 GAP in adhesion maturation of migratory CHO.K1 cells as well as synapse maturation in neurons.

### GDI-mediated attenuation of RhoGTPase signaling maintains the mature mushroom-shape spine

**Fig 6:**
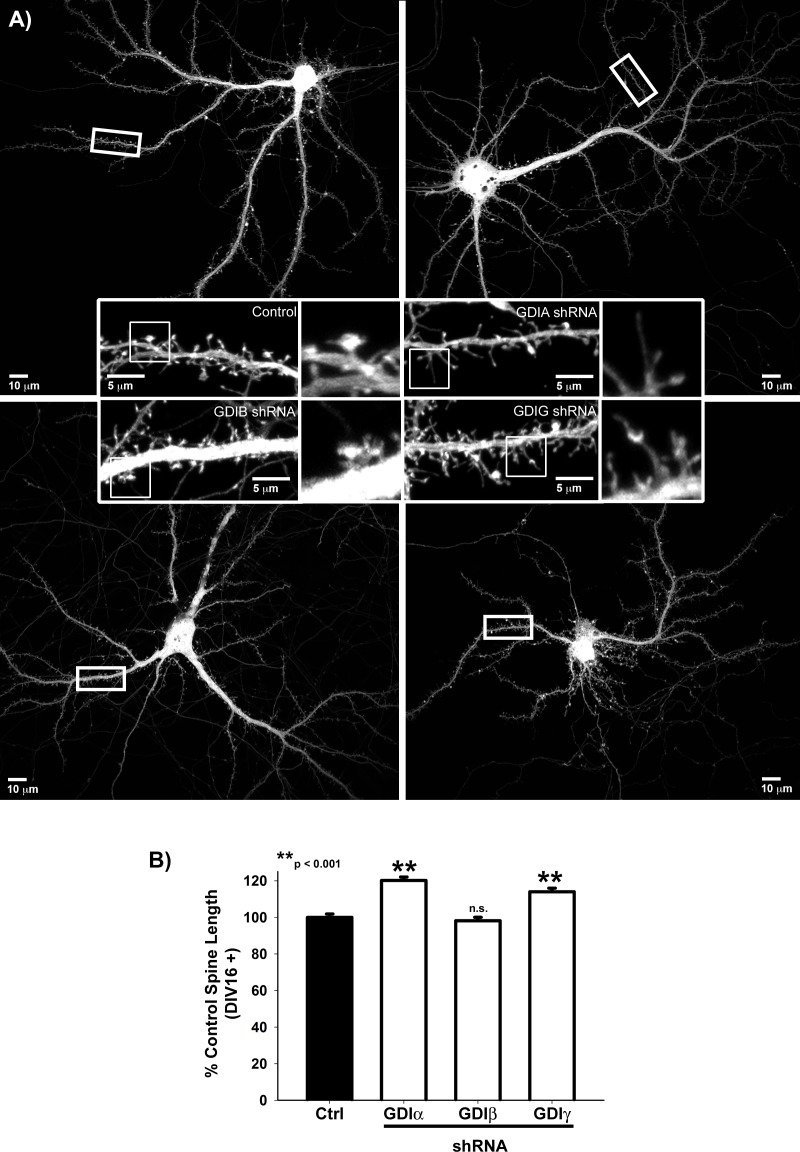
**A & B) RhoGDIα and** γ **maintain spine maturation.** Quantification of Spine Length in GFP-expressing DIV16-27 neurons transfected with the indicated shRNA targeting sequence**s** or control neurons. Spine Length is normalized to the average control spine length for each neuronal culture. n = 1232 control spines, 1757 *Arhgdi-*α shRNA spines, 1305 *Arhgdi-*β shRNA spines, and 1526 *Arhgdi-*γ shRNA spines; p < 0.001 for control neurons vs either *Arhgdi-*α shRNA or *Arhgdi-*γ shRNA neurons, but is not statistically different from *Arhgdi-*β shRNA neurons (Mann-Whitney Rank Sum Test).

We next investigated the role of the three mammalian GDI isoforms (α, β, and γ) in spine development [25]. Expression of both α and γ significantly increases during neuronal maturation as measured by real-time RT-PCR, while β exhibited negligible RNA expression during both early (DIV7) and later (DIV14) neuronal development (Fig 1B). Consistent with these RNA expression profiles, knockdown of either GDI-α or -γ, but not β, during neuronal maturation resulted in a striking reversion to immature filopodia-like spine precursors (Fig 6), demonstrating that attenuation of RhoGTPase signaling is necessary for the maintenance of mature-mushroom-shaped spines, and is the first evidence of a role for GDIs in post-synaptic spine plasticity.

## Discussion

Our research demonstrates that unique combinations of Rho GTPase family members regulate distinct stages of synaptic development. Altered spine formation and maturation are increasingly implicated in neurodevelopmental disease pathology, whereas the maintenance of mature synaptic connections is lost in neurodegenerative disease pathology. In addition, regulatory pathways of the actin cytoskeleton are increasingly implicated in these neuronal disorders. Despite this association, how synaptic connections are temporally constructed and maintained has been unclear.

We showed that distinct RhoGTPase regulators sequentially orchestrate synapse formation and maturation. During spine formation, the Rac1 GEF β-PIX promoted spine precursor formation, while the RhoA GAP OLIGOPHRENIN-1 and Cdc42 GEF FRABIN cooperatively regulated spine precursor elongation. These results lead us to hypothesize that Rac1 initiates spine precursor formation, while competition between RhoA-mediated myosin II activation and Cdc42-driven actin polymerization determines spine length. In support of this hypothesis, acute Rac1 photoactivation drove spine precursor formation, but did not affect spine length. By comparison, myosin II inhibition promoted Cdc42 activation together with increased spine length. How can we understand these differential roles for Rac1 and Cdc42-mediated actin polymerization on spine precursor formation and subsequent elongation? In general, Rac1 drives Arp2/3-mediated branched actin arrays, generating lamellipodia-like veils along the dendritic shafts of neurons [8,10,46]. Intriguingly, EM of filopodia-like spine precursors suggests that they emanate from patches of branched actin [47] consistent with a role for Rac1 in the initiation of spine precursor formation. In contrast to these branched actin structures, Cdc42 promotes linear actin polymerization, generating filopodia in migratory cells, though the role for Cdc42 in synaptic plasticity and filopodia-like spine precursors is less clear. Here we show that, while not required for spine precursor formation, FRABIN-mediated Cdc42 activation drives spine precursor elongation. This spine autonomous role for Cdc42 is consistent with recent evidence for spine- restricted Cdc42 activity in response to glutamate-mediated stimulation [48]. However, active Rac and RhoA spread through the dendrite into adjacent spines [48], suggesting either differential diffusion properties of RhoGTPases themselves or association of upstream regulators with distinct synaptic signaling scaffolds.

In contrast to spine formation, spine maturation was characterized by Rac1 inactivation via the novel RhoGTPase regulator, ARHGAP23. RhoGDI-mediated attenuation of RhoGTPase signaling pathways and subsequent actomyosin dynamics was also necessary for the maintenance of mature spines. These studies support a model for spine development whereby the switch from GEF-mediated Rac1/Cdc42 activation to GAP/GDI-mediated Rac1/RhoGTPase inactivation promotes spine maturation and the maintenance of excitatory synaptic connections. This Rac1 activity switch is consistent with previous work demonstrating that antagonism between the Rac1 GEF, TIAM1, and Rac1 GAP, BCR, control spine and synapse development [49]. Similarly, the Rac1 GEF, kalirin-7, is known to have reduced GEF activity when associated with the post-synaptic density [50], suggesting that maintenance of mature synapses involves Rac1 inactivation.

However, subsequent Rac1 and Cdc42 activation is necessary for spine head enlargement following excitatory neurotransmission [11,48]. Thus, it will be important to understand the mechanisms that transiently promote Rac1 and Cdc42 activity in response to excitatory stimulation, leading to synaptic strengthening. Consistent with the role of Rac1 and Cdc42 in synaptic strengthening, chronic Rac1 or Cdc42 inactivation with dominant negative constructs reduces spine maturation [51]. Additionally, the Rac1 GAP, ARHGAP12, negatively regulates spine volume [52], supporting the hypothesis that specific RhoGTPase regulators orchestrate distinct stages of synaptic development and plasticity. Finally, glutamate uncaging leads to coincident activation of RhoA, Rac, and Cdc42 [48], suggesting that the GDI-mediated attenuation of RhoGTPase activity must also be transiently and dynamically regulated in response to excitatory stimulation to allow for structural synaptic plasticity. Thus, it will be of interest to perform a future screen with acute knockdown followed by NMDA receptor activation to assess the role of specific RhoGTPase regulators in response to excitatory stimulation leading to synaptic strengthening. Ultimately, our identification of distinct RhoGTPase regulators for particular stages of synaptic development suggests the feasibility of targeted intervention of actomyosin dynamics during critical periods of neuronal development.

## Materials and Methods

### Antibodies and reagents

A mouse monoclonal antibody (MAB378) against the dendritic marker, MAP-2, was purchased from Millipore. A mouse monoclonal antibody against paxillin (Clone 349) was purchased from BD Biosciences. Alexa-conjugated secondary antibodies were from Invitrogen. Rhodamine phalloidin was purchased from Cytoskeleton (Denver, CO) and used at a ratio of 1:100. Blebbistatin, was purchased from Calbiochem (La Jolla, CA) and used at a concentration of 50 μM.

### Neuronal culture and transfection

Low-density hippocampal cultures were prepared from E19 rat embryos as described previously [57]. All experiments were carried out in compliance with the Guide for the Care and Use of Laboratory Animals of the National Institutes of Health and approved by the University of Virginia Animal Care and Use Committee (Protocol Number: 2884). Neurons were plated on glass coverslips coated with 1 mg/ml poly-L-lysine at an approximate density of 70 cells/mm^2^ and were transfected at DIV-6/7 using a modified calcium phosphate precipitation method as described previously [57] or at DIV-14 with Lipofectamine 2000 (Life Technologies) used at a ratio of 1.3 μl lipofectamine 2000 per 1 μg DNA. shRNAs were co-transfected with GFP. Neurons were fixed and stained 48–72 hours after transfection.

### Plasmids and shRNA sequences

Target small hairpin RNA (shRNA) sequences used in this study were cloned into a pSuper vector (Oliogoengine) between the *Bam*HI and *Hin*dIII restriction sites.

*Arhgap23* shRNA #1: 5´-CATCGAGGCCAATCGGATA-3´

*Arhgap23* shRNA #2: 5´-CCGCAGAGGATCATCGTGA-3’

*Arhgdia* shRNA #1: 5’-GGTGTGGAGTACCGGATAA-3’

*Arhgdib* shRNA #1: 5’-GCTAAAGGAAGGTATCGAA-3’

*Arhgdig* shRNA: 5’-ACGAGGTGCTGGACGAAAT-3’

*Arhgef7 (*β*-pix)* shRNA #1: 5’-CAACAGGACTTGCACGAAT-3’

*Arhgef7 (*β*-pix)* shRNA #2: 5’-GGAGCATGATCGAGCGCAT-3’

*Arhgef9* shRNA #1: 5’-CAACAAGGAAACCGAAGAA-3’

*Arhgef9* shRNA #2: 5’-ACATAGACCTTTACGTATA-3’

*Frabin* shRNA #1: 5’-CCAAGATGGATACGTGATA-3’

*Frabin* shRNA #2: 5’–GTAAATATCCCCAGCGGTA-3’

*Oligophrenin-1* shRNA #1: 5’-TGAGATTAATATTGCGGAA-3’

*Oligophrenin-1* shRNA #1: 5’-CCAGTCGTTTCAGTTTGAT-3’

*Vav2* shRNA #1: 5’-GGTGGAAGGGCGAGACGAA-3’

*Vav2* shRNA #2: 5’-CCCAGTTCCTGTGTCTGAA-3’

Full-length human *Arhgap23* was obtained from Dharmacon ORFeome library and subcloned into the pEGFP-N1 vector (Clonetech). FRET biosensor probes, including Raichu Rac Cdc42 and WT Raichu Rac and constitutively active, Raichu Rac G12V (V12), were kindly provided by M. Matsuda (Osaka University, Osaka, Japan) [39], and consist of YFP, the CRIB domain of human PAK1 (amino acids 68–150), human Cdc42 or Rac1 (amino acids 1–176) and CFP cloned into a derivative of the pCAGGS vector [91]. The genetically encoded photoactivatable Rac was kindly provided by Dr. Klaus Hahn (UNC Chapel Hill, NC, USA) [36], and was activated by exposure to room lighting for 10min (Fig 1C) or acute activation with 458nm light for approximately 1 min using confocal imaging as described below. Full-length chicken paxillin-GFP cloned into the pEGFP-N3 vector (Clontech) has been described previously [92] and GFP was replaced by mCherry from R. Tsien (University of San Diego, San Diego, CA) [93].

### Immunocytochemistry

Neurons and CHO.K1 cells were fixed in 4% formaldehyde ultra-pure EM grade (Polysciences, Inc., Warrington, PA) + 4% sucrose in PBS for 20 min at room temperature and permeabilized with 0.2% Triton X-100 for 5–10 min at room temperature, followed by blocking and antibody incubations in 5–20% normal goat serum (Abcam). Coverslips were mounted with Vectashield mounting media (Vector Laboratories, Burlingame, CA).

### CHO.K1 cell culture and transfection

Wild-type CHO.K1 fibroblast were cultured in low glucose DMEM (Invitrogen/Life Technology, Invitrogen) supplemented with nonessential amino acids, 100 units/ml penicillin, 100 μg/ml streptomycin, and 10% FBS. Co-transfections of Raichu-Rac FRET probes and plasmids containing the ArhGAP23 shRNAs were perform using Lipofectamine 2000 (Invitrogen) at a ratio of 5 μl lipofectamine per 1 μg DNA. After 48–72 hours, 1X10^4^-2x10^4^ cells were plated for 2–4 hours on glass bottom dishes, which were coated with 2-μg/ml fibronectin. For FRET, cells were either imaged live or fixed by incubation for 15–20 minutes at RT with a 4% paraformaldehyde + 4% sucrose PBS solution and imaged in PBS.

### Quantitative real-time PCR (qRT-PCR)

Total RNA was extracted from cells with TRIZOL (Invitrogen). Total RNA was reverse transcribed using the iScript CDNA Synthesis Kit (BioRad). qRT-PCR assays were performed using TaqMan Gene Expression Assays (Applied Biosystems) according to manufacturer´s protocol. The following FAM-MGB TaqMan probes (Applied Biosystems/ThermoFisher) were used to analyze expression of rat RhoGTPase regulators during neuronal development: Rat *Actb* (Rn00667869_m1); Rat *Arhgap23* (Rn01496413_m1); Rat *Arhgdia* (Rn01751927_g1); Rat *Arhgdib* (Rn01459333_m1); Rat *Arhgdig* (Rn01750774_m1); Rat *Arhgef7 (*β*-pix*) (Rn00586980_m1); Rat *Arhgef9* (Rn00576661_m1); Rat *Fgd4* (*Frabin*) (Rn00594991_m1); Rat *Oligophrenin-1* (Rn01752886_m1); Rat *Vav2* (Rn01436349_m1)

PCR reactions were run using a StepOnePlus Real-Time PCR System (Applied Biosystems, USA) under the following thermocycler conditions of 95°C for 10 min, 40 cycles of 95°C for 30 sec, 58°C for 1 min and 72°C for 1 min. All samples were tested in triplicate and mean Ct values were calculated for transcriptional changes by normalizing to a standard curve for the housekeeping gene, human beta actin (Hs01060665 probe). No-template controls were also included in each amplification run to monitor for contamination.

### Confocal imaging and analysis

Confocal images were acquired on a laser scanning Olympus Fluoview 1000 microscope (IX81 base) equipped with a 60X/1.35 NA oil objective (Olympus). Green fluorescent probes (GFP) were excited with a 488 nm laser line of a multi-Argon laser, while red probes (Rhodamine) were excited with the 543 nm laser line of a He-Ne laser; and the far-red probe Alexa647 was excited with the 635 nm line of a diode laser. Fluorescence emission was collected using the following dichroic mirror/filter combinations: SDM560/BA505-525 (GFP), SDM640/BA560-620 (mCherry, RFP, Alexa568 and Rhodamine) and BA655-755 (Alexa647). Fluorescent images were collected in a Z-stack and in sequential line scanning mode using Olympus Fluoview software. Image analysis was performed with Image J software on max intensity Z projections.

### Statistical analysis

Statistical analysis was performed using Sigma Plot 11.0. In each case, we first assessed whether the data assumed a normalized distribution using the Shapiro-Wilk test and also if the two data sets under comparison presented with equal variance. If the data assumed a normalized distribution with equal variance, the t-test was used to assess statistical significance. However, if the data failed to have either a normalized distribution or equal variance, we used a non-parametric statistical analysis, the Mann-Whitney Rank Sum Test. We have indicated the test used to determine statistical significance in each figure legend.

### FRET imaging and analysis

Confocal images were acquired with a Fluoview 1000 microscope using a 60× 1.35 NA oil objective (Olympus). For dual-emission ratio imaging of Raichu-Cdc42/Rac, we used the 458 nm line of a multi-Argon ion laser for CFP excitation and SDM510/BA480-495 (CFP) and BA535-565 (YFP/FRET) filters for the collection of CFP and YFP fluorescence emission, respectively. Ratiometric FRET images of max intensity Z projections of the acquired CFP and YFP images were created using the Biosensor Processing Software 2.1 available from the Danuser laboratory (University of Texas Southwestern, Dallas, TX). Image analysis of the resulting FRET images was performed with Image J software.

### Total Internal reflection microscopy (TIRF)

TIRF images were acquired with an Olympus inverted microscope (IX70) fitted with a Ludl modular automation controller (Ludl Electronic Products, Hawthorne, NY) using an Olympus 60X/1.45NA oil Plan Apochromatic TIRFM objective and charge-coupled device (CCD) camera (Retiga Exi, QImaging). CHO.K1 cells were plated on 2ug/ml fibronectin-coated glass coverslips and imaged in HyClone CCM1 media (GE Healthcare) at 37°C within ~1hour of plating. Images were acquired with Metamorph Imaging Software (Molecular Devises, Sunnyvale, CA). Green fluorescent probes (GFP) were excited with a 488 nm laser line of a multi-Argon laser, while red probes were excited with a 561nm diode laser. Images were acquired every 5 sec for a total of 10 min.

## Supporting Information

S1 Fig Validation of shRNAs expressed in REF-52 cells. Protein schematics are based on information available from www.uniprot.org, and the corresponding region targeted by the different shRNAs are depicted below the schematic. Note: We were unable to detect expression of either *Fdg4* (*Frabin*) or *Arhgdig* (RhoGDI-γ) in either REF-52 or CHO.K1 cell lines. However, the two different shRNAs targeting FDG4 did not downregulate expression of the other GEFs, either β*-pix* or *Vav2*, and both shRNAs similarly reduced spine length (Fig 2D), and the involvement of Cdc42 regulation in spine length was further confirmed by a FRET biosensor (Fig 2E). *Arhgdig*, which was upregulated during neuronal development similar to *Arhgdia* (Fig 1B), similarly affected mature spine morphology (Fig 5).
(TIF)

S1 Video Rac photoactivation promotes spine precursor formation. A DIV-14 primary rat hippocampal neuron transfected with a genetically encoded photoactivatable Rac was exposed to 458 nm light at the indicated region of photoactivation at t = 0 sec (continuous slow scan speed for ~49 sec), and the resulting spine precursor formation was monitored by timelapse confocal microscopy. The magenta reference image represents neuron morphology at the time of photoactivation and is superimposed with GFP timelapse images before (t = -120 sec to 0 sec) and after photoactivation (t = 0 sec to 300 sec).
(AVI)

S2 Video Adhesion maturation in a control CHO.K1 cell. A CHO.K1 cell transfected with paxillin mCherry was plated on fibronectin and imaged by TIRF microscopy to visualize adhesions. In the bottom right, a protrusion exhibits small nascent adhesions, some of which begin to mature into focal adhesions. Each frame = 10 sec for a total of 10 min.
(AVI)

S3 Video ARHGAP23 promotes adhesion maturation. A CHO.K1 cell transfected with paxillin mCherry and ARHGAP23-GFP was plated on fibronectin and imaged by TIRF microscopy to visualize adhesions. GAP23 results in elongated adhesions, particularly along the side of the protrusion. Each frame = 10 sec for a total of 10 min.
(AVI)
