# Supplementary figures and images for: RhoGTPase Regulators Orchestrate Distinct Stages of Synaptic Development
Source: PLoS One. 2017 Jan 23;12(1):e0170464. doi: 10.1371/journal.pone.0170464 (PMC5256999; doi:10.1371/journal.pone.0170464)

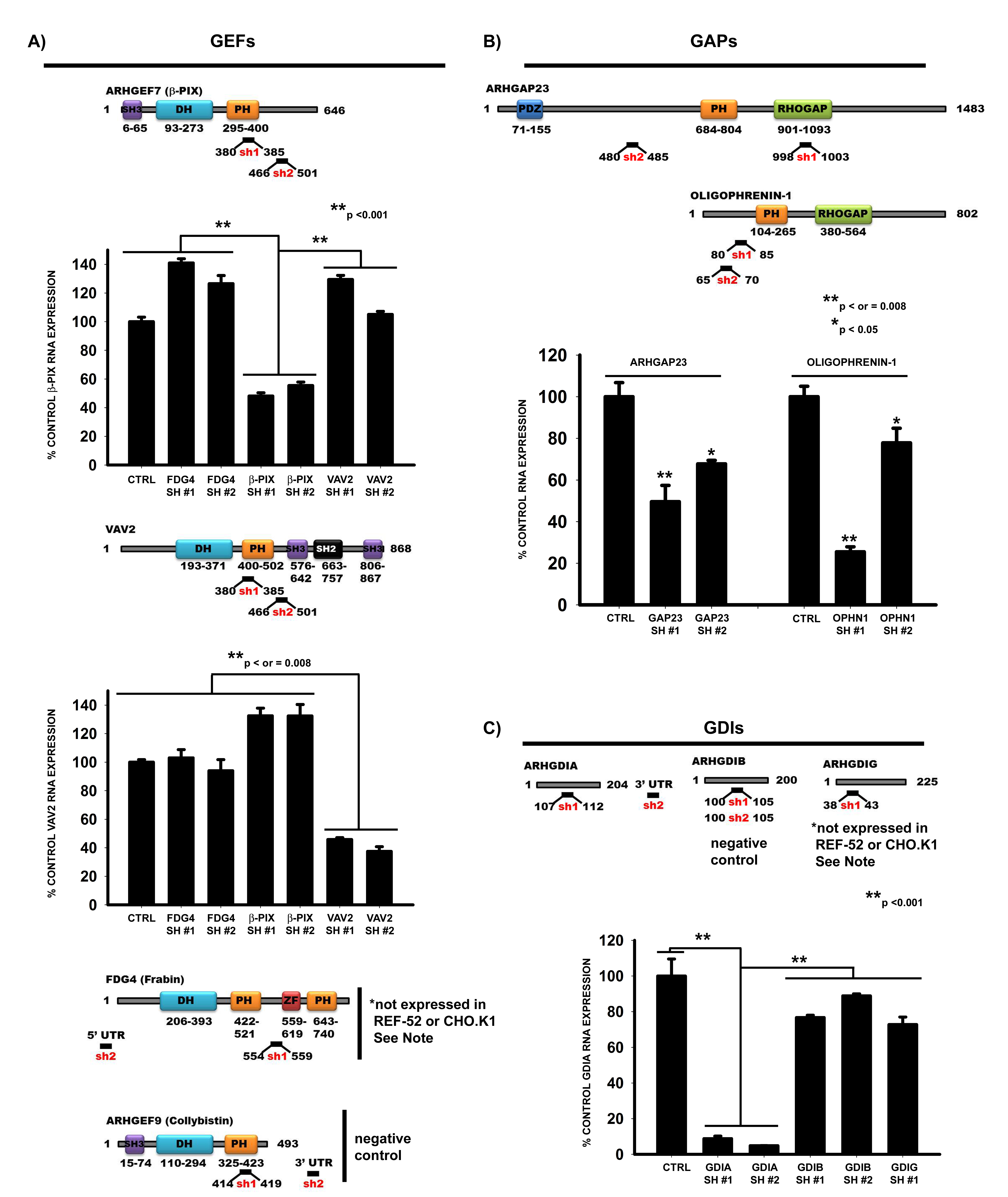

Supplement: S1 Fig — Protein schematics are based on information available from www.uniprot.org, and the corresponding region targeted by the different shRNAs are depicted below the schematic. Note: We were unable to detect expression of either Fdg4 (Frabin) or Arhgdig (RhoGDI-γ) in either REF-52 or CHO.K1 cell lines. However, the two different shRNAs targeting FDG4 did not downregulate expression of the other GEFs, either β-pix or Vav2, and both shRNAs similarly reduced spine length (Fig 2D), and the involvement of Cdc42 regulation in spine length was further confirmed by a FRET biosensor (Fig 2E). Arhgdig, which was upregulated during neuronal development similar to Arhgdia (Fig 1B), similarly affected mature spine morphology (Fig 5). (TIF) [file pone.0170464.s001.tif]
